# Supplementary material for: Miro1‐dependent mitochondrial positioning drives the rescaling of presynaptic Ca2+ signals during homeostatic plasticity
Source: EMBO Rep. 2016 Dec 30;18(2):231–40. doi: 10.15252/embr.201642710 (PMC5286383; doi:10.15252/embr.201642710)
Supplement: Supplementary file 1 — Expanded View Figures PDF [file EMBR-18-231-s001.pdf]

Expanded View Figures

Figure EV1. SyGCaMP5 is localised to presynaptic terminals and not saturated during 10 Hz stimulation.

A Diagram representing the SyGCaMP5 vector.

B, C Hippocampal neurons transfected with SyGCaMP5 (green) and immunostained with anti-SV2 (B) or Piccolo (C) to label presynaptic terminals. Arrowheads indicate SyGCaMP5 clusters positive for Piccolo. Scale bars: 10 μm (B), 5 μm (C).

D Hippocampal neurons transfected with SyGCaMP5 and immunostained with tau to label the axon and MAP2 to label the somato-dendritic region. Scale bar, 30 μm. (i, ii) High-magnification images of the cell body (i) and axon (ii), verifying that the construct SyGCaMP5 is targeted to presynaptic sites. Scale bar, 10 μm.

E Example trace of a hippocampal neuron transfected with SyGCaMP5. During time points 20–30 s, 10-Hz field stimulation (10 V) was applied followed by high KCl treatment (50 mM KCl).

F Summary bar graph represents the average fluorescence during stimulation (between time points 20 and 30 s, $\Delta F/F_0$ = 1.7 ± 0.2) and the average fluorescence during the first five frames of KCl treatment ($\Delta F/F_0$ = 2.8 ± 0.4, $^{**}P < 0.001$, paired t-test, n = 3 neurons).

Data information: Experiments were performed in E18 and P0 rat hippocampal neuronal cultures at DIV 8–10. Error bars represent SEM.

© 2016 The Authors

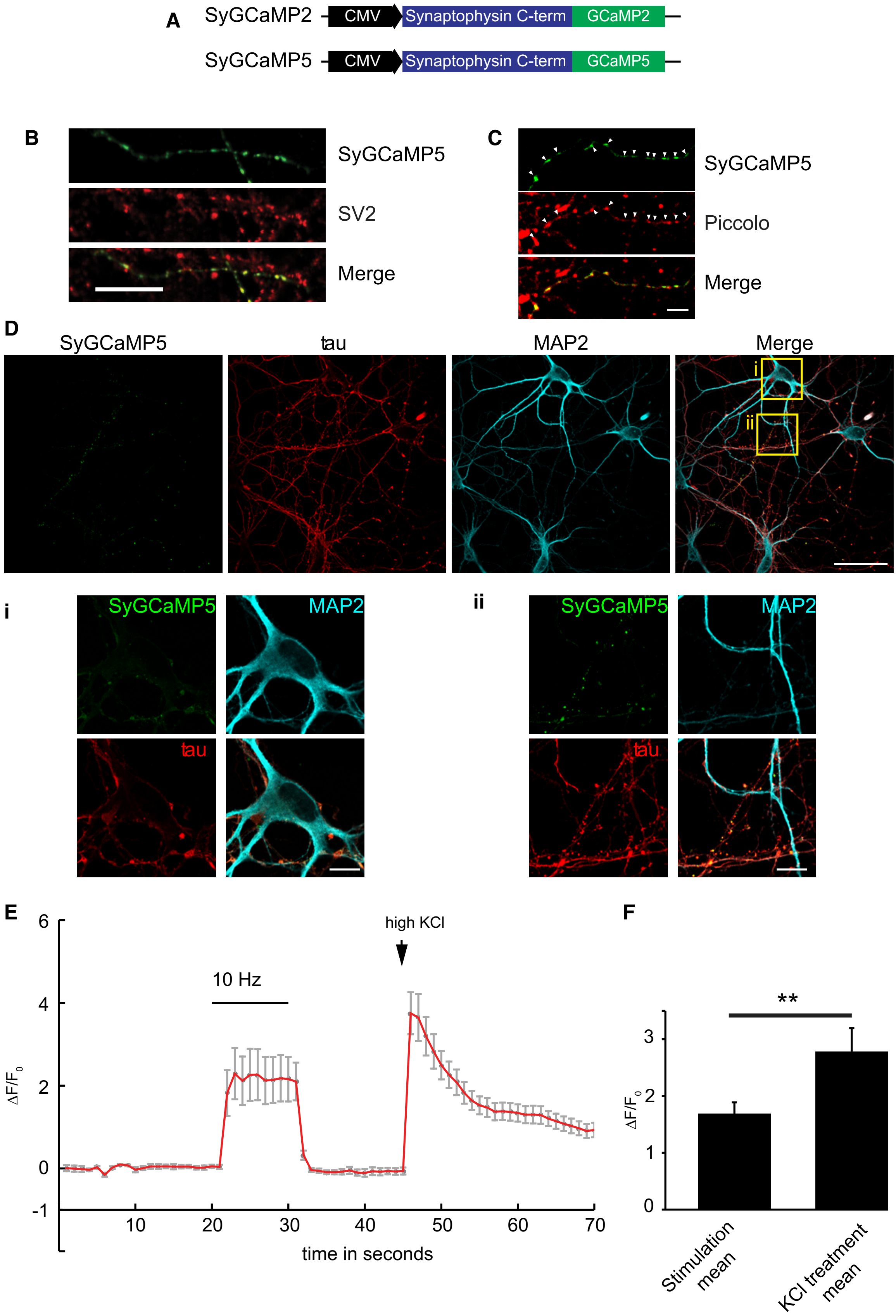

Figure EV1.

 © 2016 The Authors

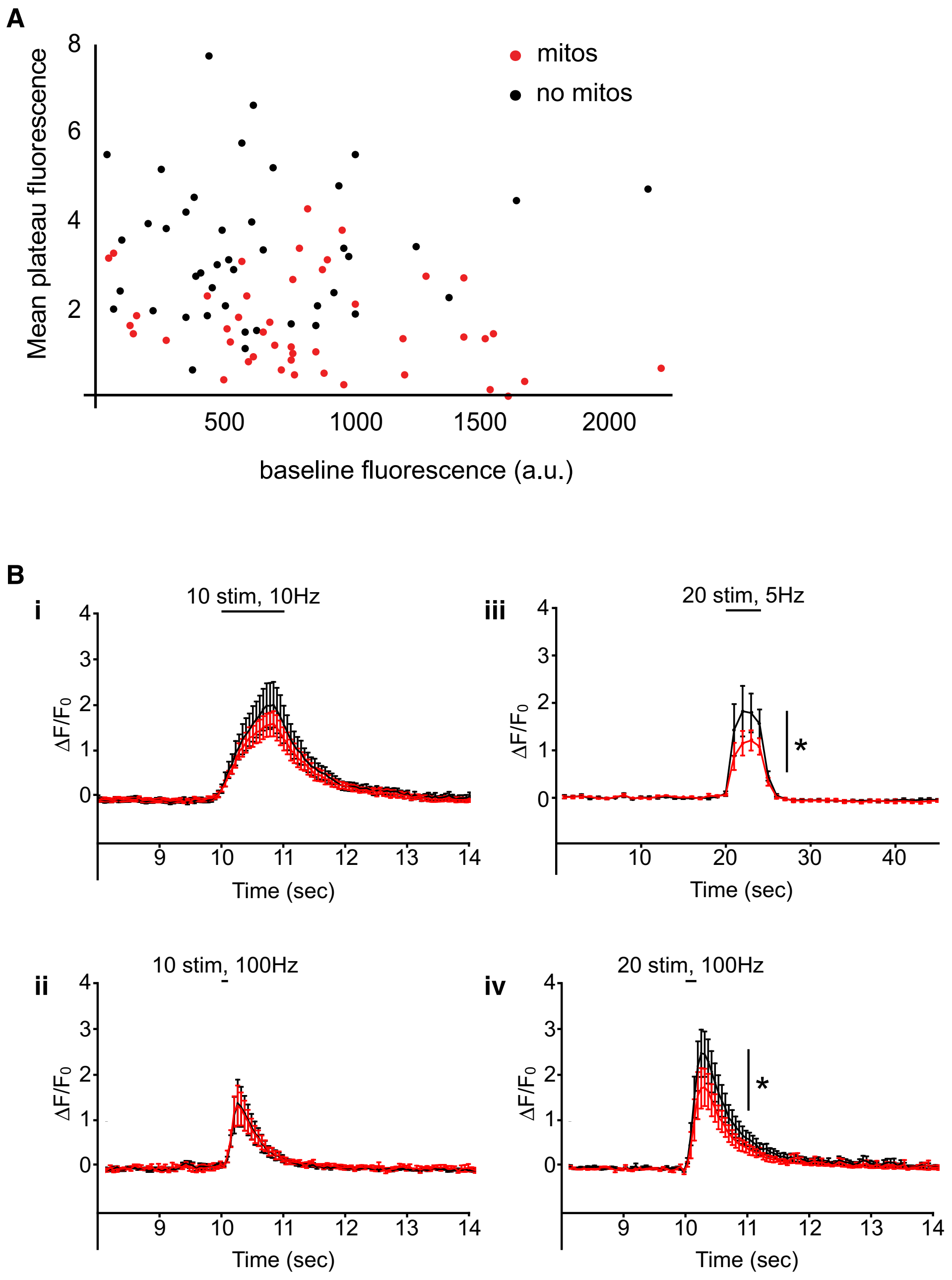

Figure EV2. SyGCaMP5 baseline fluorescence does not correlate with $\Delta F/F_0$ reached and number of stimuli, not frequency, determines mitochondrial modulation of presynaptic Ca^{2+} response.

A Scatterplot of the mean plateau fluorescence of SyGCaMP5 that is reached, plotted against the baseline fluorescence (average of time points 1–20 s) of each terminal analysed. Experiments were performed in P0 rat hippocampal neuronal cultures at DIV 10–12.

B Average $\Delta F/F_0$ SyGCaMP5 traces for 10 stimuli delivered at (i) 10 Hz (recorded at maximum frames per second, n = 13 neurons, 44 terminals, $\Delta F/F_0$ = 1.0 ± 0.2 with and 1.2 ± 0.3 without mitochondria, paired t-test, P = 0.68) and (ii) 100 Hz (recorded at maximum frames per second, n = 4 neurons, 18 terminals, $\Delta F/F_0$ = 0.8 ± 0.2 with and 0.8 ± 0.2 without mitochondria, paired t-test, P = 0.60) did not demonstrate differences between synapses with and without mitochondria. In contrast, differences were observed when 20 stimuli were delivered, whether at (iii) 5 Hz (recorded at 1 frame per second, n = 11 neurons, 58 terminals, $\Delta F/F_0$ = 0.9 ± 0.2 with and 1.3 ± 0.4 without mitochondria, paired t-test, *P < 0.05) or (iv) 100 Hz (recorded at maximum frames per second, n = 12 neurons, 87 terminals, $\Delta F/F_0$ = 1.2 ± 0.3 with and 1.7 ± 0.4 without mitochondria, paired t-test, *P < 0.05). Experiments were performed in E18 rat hippocampal neuronal cultures at DIV 10–12. Error bars represent SEM.

© 2016 The Authors

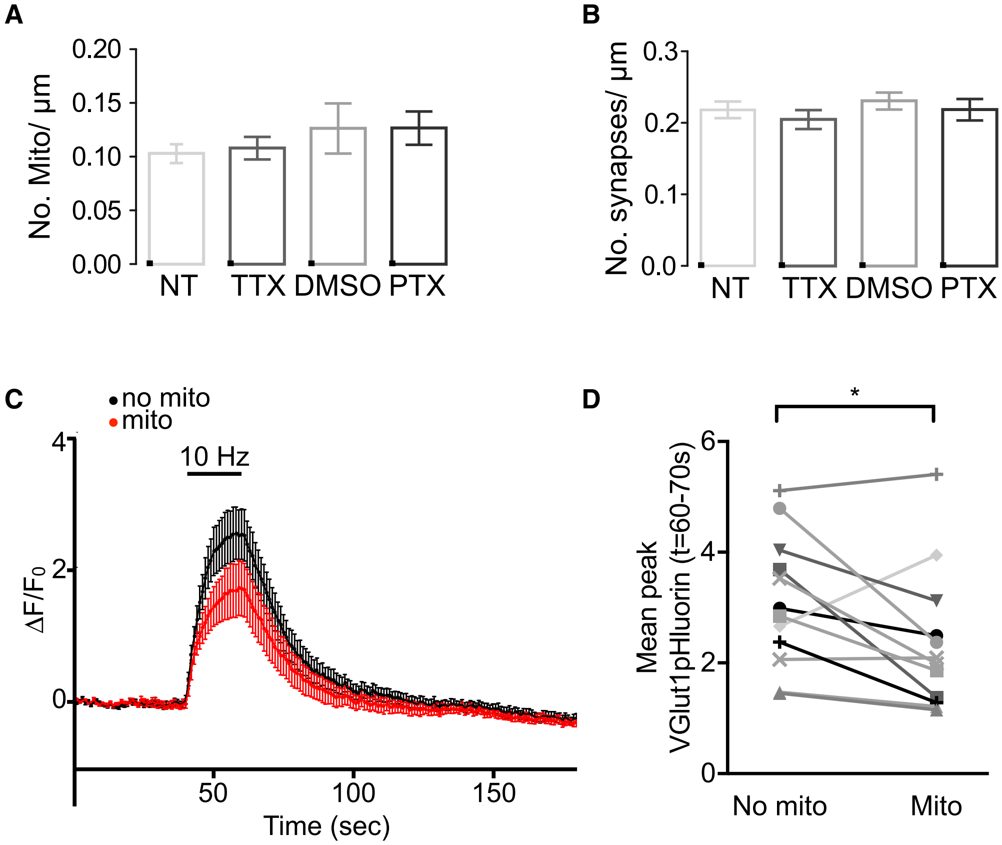

Figure EV3. Quantity of axonal mitochondria and synaptic boutons is unchanged following homeostatic plasticity, and mitochondrial modulation of presynaptic vesicular release is maintained following picrotoxin treatment.

A, B Quantification of the number of mitochondria (A) and SYN-GFP clusters (B) per µm in control versus TTX- and PTX-treated axons (n = 12 axons, ANOVA $P > 0.05$).

C, D Average traces of presynaptic terminals of hippocampal neurons transfected with VGlut1pHluorin and Mito-mKate2 after 48 h of treatment with picrotoxin and stimulated using field stimulation at 10 Hz for 20 s (40–60 s). Terminals with a mitochondrion are represented by the red trace, and terminals without are represented by the black trace. (D) Summary graph corresponds to the traces shown in panel (C). Mean stimulation fluorescence (time points 60–70 s) is compared in terminals of the same neuron which contain mitochondria (right, $\Delta F/F_0 = 2.4 \pm 0.4$) compared to terminals without mitochondria (left, $\Delta F/F_0 = 3.1 \pm 0.3$) ($n$ = 12 neurons, 58 terminals, $*P < 0.05$, paired t-test).

Data information: Experiments were performed in E18 rat hippocampal neuronal cultures at DIV 10–12. Error bars represent SEM.

Figure EV4. Co-transfection of MtDsRed and ΔEF-Miro1-myc/Miro1-myc does not affect the localisation of synapses.

A, B Example images of hippocampal neurons used for analysis in Fig 3. Neurons transfected with ΔEF-Miro1-myc (A) or Miro1-myc (B) are co-stained with anti-myc antibody.

C Hippocampal neurons transfected with MtDsRed alone and stained with anti-myc antibody. It can be seen that staining with anti-myc did not label the MtDsRed-transfected neuron. Scale bar, 40 µm.

D Hippocampal neurons transfected with SYN-GFP, MtDsRed, and Miro1 or ΔEF-Miro1 and immunostained with Piccolo to label presynaptic terminals. Arrowheads indicate SYN-GFP clusters that are positive for Piccolo. Scale bar, 5 µm.

E Quantification of the density of SYN-GFP clusters within the axon of control neurons expressing MtDsRed alone, or those expressing Miro1 or ΔEF-Miro1 (processes: control n = 42, Miro1 n = 28, ΔEF-Miro1 n = 33, ANOVA ns).

F–H Fraction of SYN-GFP clusters co-localising with mitochondria, following postsynaptic glutamate receptor block (APV: 100 µM, NBQX: 10 µM, 24 h) in (F) control conditions (NT: 0.302 ± 0.02, n = 43; APV + NBQX: 0.214 ± 0.02, n = 36; t-test, $**P < 0.01$), (G) with the expression of Miro1 (NT: 0.315 ± 0.03, n = 26; APV + NBQX: 0.287 ± 0.03, n = 25; t-test ns) and (H) with the expression of ΔEF-Miro1 (NT: 0.256 ± 0.03, n = 24; APV + NBQX: 0.259 ± 0.05, n = 26; t-test ns).

Data information: Experiments were performed in E16 mouse and E18 rat hippocampal neuronal cultures at DIV 10–12. Error bars represent SEM.

© 2016 The Authors

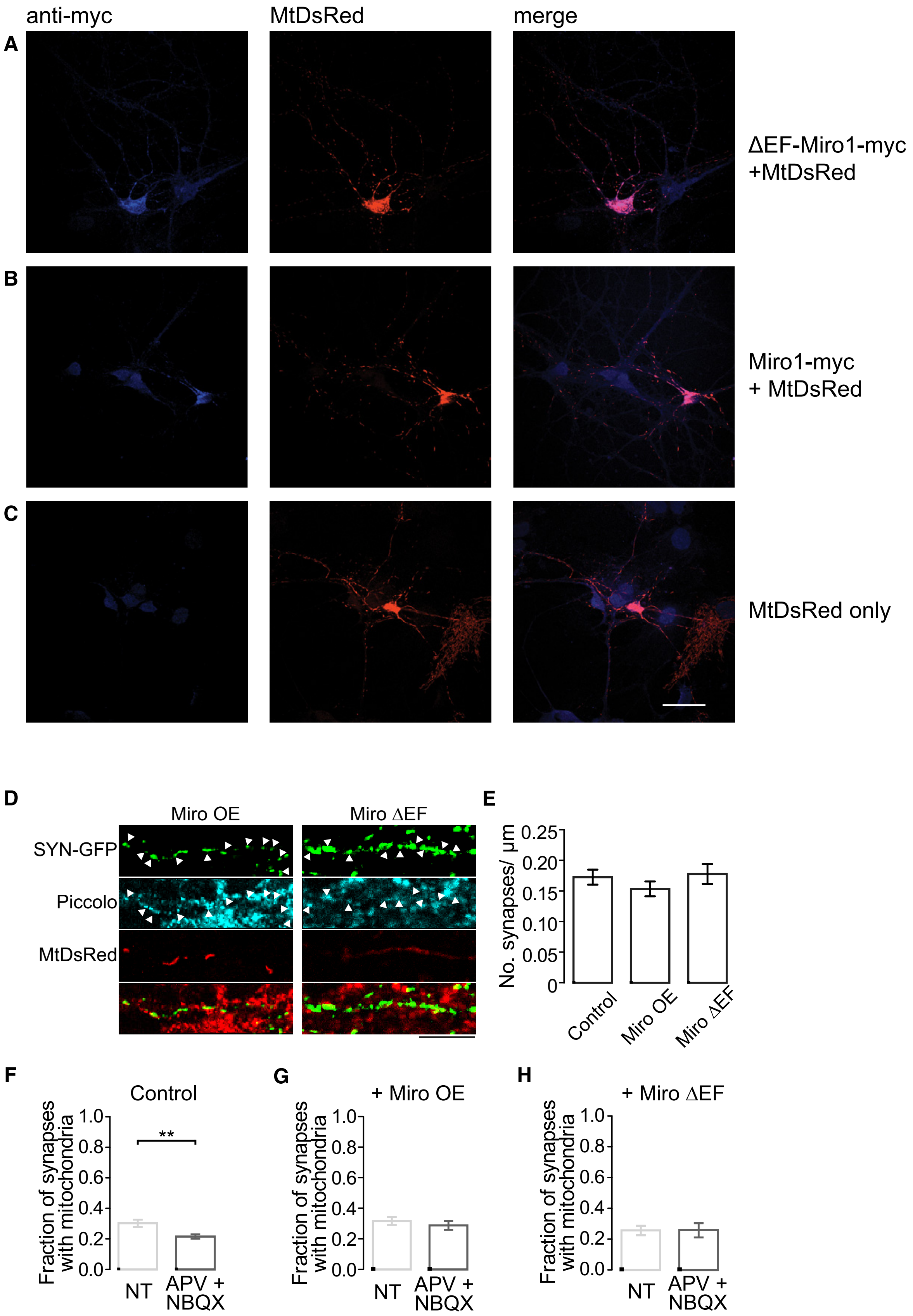
anti-myc
MtDsRed
merge
A
ΔEF-Miro1-myc
+MtDsRed
B
Miro1-myc
+ MtDsRed
C
MtDsRed only
D
Miro OE
Miro ΔEF
SYN-GFP
Piccolo
MtDsRed
E
No. synapses/ µm
0.25
0.20
0.15
0.10
0.05
0
Control
Miro OE
Miro ΔEF
F
Control
**
G
+ Miro OE
H
+ Miro ΔEF
Fraction of synapses with mitochondria
1.0
0.8
0.6
0.4
0.2
0.0
NT
APV + NBQX

Figure EV4.

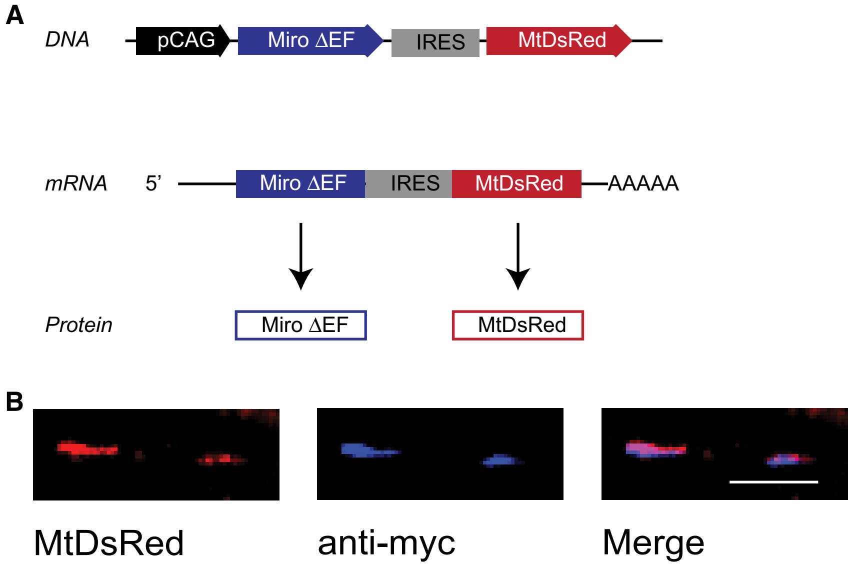

Figure EV5. IRES construct expresses both MtDsRed and ΔEF-Miro1-myc.

A Diagram of the IRES vector, which enables bicistronic expression of MtDsRed and ΔEF-Miro1-myc as it contains an internal ribosome entry site (IRES).

B Mitochondria labelled with MtDsRed are also stained with anti-myc antibody. Scale bar, 10 μm.

 © 2016 The Authors
